# Status of Resistant and Knockdown of West Nile Vector*, Culex pipiens* Complex to Different Pesticides in Iran

**Published:** 2019-09-30

**Authors:** Sara Rahimi, Hassan Vatandoost, Mohammad Reza Abai, Ahmad Raeisi, Ahmad Ali Hanafi-Bojd

**Affiliations:** 1 Department of Medical Entomology and Vector Control, School of Public Health, Tehran University of Medical Sciences, Tehran, Iran; 2 Department of Environmental, Chemical Pollutants and Pesticides, Institute for Environmental Research, Tehran University of Medical Sciences, Tehran, Iran; 3 National Program Manager for Malaria Control, Center for Communicable Diseases, Ministry of Health and Medical Education, Tehran, Iran

**Keywords:** Resistance, *Culex pipiens*, West nile, Insecticide, Iran

## Abstract

**Background:**

West Nile virus (WNV) can cause a fatal disease in humans and it is mainly transmitted to people through the bites of infected mosquitoes. Vector control using insecticides is a very important goal. Study of *Culex pipiens* resistance towards several insecticides in the city of Tehran, Iran was evaluated.

**Methods:**

Adult females reared from field-caught larvae from southern part of Tehran and lab strain reared in the insectary of Tehran University of Medical Science were determined for resistant status by exposing to 4% DDT, 0.1% bendiocarb, 0.1% propoxur, 1% fenitrothion, 0.05% deltamethrin, 0.75% permethrin, 0.05% lambda-cyhalothrin, 0.5% etofenprox, 5% malathion and 0.15% cyfluthrin papers using the standard WHO susceptibility tests.

**Results:**

Results clearly showed resistance development of *Cx. pipiens* against tested insecticides. Mortalities of *Cx. pipiens* were less than 90% with high resistance, low knock down rate and knock down time (50%) observed against insecticides. DDT and Malathion showed the most and least lethal time (LT_50_) values for the field strain. The results of the knockdown test showed that DDT and deltamethrin had the most and least knockdown times (50%) for the field strain, respectively, while DDT and lambda-cyhalothrin had the most and least knockdown times (50%) for the lab strain, respectively.

**Conclusion:**

Resistance to mentioned insecticides in *Cx. pipiens* is widely distributed in southern part of Tehran. Regular implementation of susceptibility test in *Cx. pipiens* mosquitoes will help local public health authorities to develop new and better control strategies.

## Introduction

*Culex pipiens* is a one of the most important vectors of disease in humans and animals. These mosquitoes are the main vector of west nile viruses (WNV) disease in the world. Currently, WNV (*Flavivirus*: *Flaviviridae*) is the most widespread arbovirus in the world. WNV causes a fatal neurological disease in human and approximately 80% of people infected will not show any symptoms. Although *Cx. pipiens* does not transmit diseases in Tehran, it is the most common mosquito species in urban and rural areas of Tehran and this led to discomfort especially in urban areas (1, 11). Insecticides play a central role in controlling major vectors of diseases such as mosquitoes (2). Vectors control is facing many problems especially occur-rence of insecticide resistance. Resistance to insecticides is the most important problem impeding the execution of vector control programs. However, the heavy and long-term use of insecticides in public health and agriculture seems to drive development of insecticide resistance in *Cx. pipiens* (3). Resistance to DDT, organ-ophosphate, carbamate and pyrethroids tolerance has been documented in populations of *Cx. pipiens* from many countries (4–6), but little is known about the resistance and knock down status of this vector in Iran. The emergence of resistance phenomenon not only shortens the lifespan of existing insecticides but also obstacles to efficacy of new pesticides owing to cross-resistance and multiple resistance mechanisms (7, 8). Nearly more than 100 mosquito species are reported as resistance to one or more insecticide (9) including 56 species from *anophelinae* subfamily and 39 species from *culicinae* subfamily (10). In Iran, home sewage systems are considered as a breeding place for *Cx. pipiens.* The sewage system conducted from north to the south of capital city, Tehran, where makes a suitable places for growth and increasing of these mosquitoes. This species is considered as a main nuisance mosquito in the country. The *Cx*. *pipiens* complex is main prevalent species and bred at the sewage system and rice fields. According to Ministry of Health and Medical Education as well as Ministry of Jihad Keshavarzy of Iran, different insecticides and rodenticides are being used in the area for household and agricultural pest control. Use of such pesticides indirectly cause selection pressure on the susceptibility of mosquitoes mainly breed in waste water habitats (11–13). In Iran, *Cx. pipiens* have shown resistance against DDT, carbamate, and organophosphate insecticides (14) while they have shown some levels of tolerance to permethrin, lambdacyhalothrin, deltamethrin, and etofenprox (11). Understanding insecticide resistance mechanisms is essential for vector control strategies. Resistance mechanisms can be divided into two major groups, decreased sensitivity of the target proteins known as target site insensitivity and increased metabolic detoxification of insecticides (15). In *Cx. pipiens*, the most common target site insensitivity is the L1014Fkdr mutation in the voltage-gated sodium channel (VGSC) gene, conferring resistance to DDT and pyrethroids, followed by the G119S ace-1 mutation, conferring resistance to organophosphate and carbamate (16). Knockdown resistance (kdr) is meant the resistance of insects to pyrethroid and DDT due to structural changes created in sodium channels resulted by point mutation in sodium channel gene (17–19) which reduces the sensitivity of sodium channels and prevents the connection of insecticides and as result decreases their efficiency (20). Three major classes of enzymes, cytochrome P450 oxidases, esterases and glutathione-S-transferases (GST) have been reported to be involved in metabolic resistance to pyrethroids, organ-ophosphate and organocholine, respectively, in *Cx. pipiens* (16–19). Kdr resistance was first recognized in *Musca domestica* flies (21) and was then reported in other insects (22). Two forms of mutations (L1014F and L1014S) in *Cx. pipiens* complex that cause kdr resistance are reported (23). Considering that in Tehran metropolitan control of these mosquitoes outbreaks relies heavily on the use of insecticides however, the increasing of resistance to insecticides can significantly limit the continued application of these control agents in Iran, it is necessary to assess the current resistance status of *Cx. pipiens* for effective control programs. The objective of this study was to assess the knock down status and susceptibility of *Cx. pipiens* populations in Tehran, Iran to mentioned different adulticide to plan an effective mosquito control program.

## Materials and Methods

### Collection site and strains of *Culex pipiens*

Different stages of larvae and pupae samples of *Cx. pipiens* were collected from polluted drains or waste water containers in urban areas at the southern part of Tehran and transferred to the insectary at the department medical entomology, Tehran university of medical science, and bred the immature stages using the habitat water and a few amount of Tetramin® fish food. A laboratory strain of *Cx. pipiens* from Tehran University of medical science has been maintained in the insectary for several years.

### Laboratory colonization

Colonies were reared at 26±2 °C, 70±5% relative humidity, and 14: 10h light: dark conditions. Larvae were fed with Tetramin® fish food. Adult females were fed blood from live chickens 2 times per week. Adults had access to 10 % sucrose solution from a cotton wick until 3–5 day old female mosquitoes which used for the tests. Containers filled with deionized water were placed inside the cages for female ovi-position. Egg cages were transferred to new containers and larvae were reared after hatching.

### Insecticides

Ten different adulticides were tested. 4% DDT, 0.1% bendiocarb, 0.1% propoxur, 1% fenitrothion, 0.05% deltamethrin, 0.75% permethrin, 0.05% lambda-cyhalothrin, 0.5% etofenprox, 5% malathion and 0.15% cyfluthrin-impregnated papers were obtained from the WHO. For the control, impregnated-papers containing 1ml ethanol were used.

### Insecticide Susceptibility Test

Adult tests were determined by a bioassay using the methods recommended by the WHO. In the adult bioassay test, groups of sucrosefed 3–5 day old adult female mosquitoes per replicate were used for this bioassay. This test has used different types of impregnated papers that mentioned above. There were three replicates per bioassay including the control. Control mosquitoes were exposed to paper without insecticide. All mosquitoes used for this test were exposed to the diagnostic dosage of insecticides for 60 minutes in standard WHO test tubes, with minor modifications. Sucrose solution was provided for the mosquitoes. Knock down mosquitoes counts and knockdown time 50% (KT_50_) were recorded within the logarithm times of 8 to 256min. The test mosquitoes and the controls were held for a 24h recovery period and the mortality rate, lethal time 50% (LT_50_) recorded. Insecticide resistance status was evaluated using the classification determined by WHO: 98–100% mortality indicates susceptibility, 90–97% mortality suggests possible resistance requiring confirmation, and 90% mortality suggests resistance (24, 25).

### Data analysis

If the control mortality was between 5% and 20%, the percentage mortality was corrected by Abbott’s formula (37). Adult mortality, knock down data were analyzed with the regression-probit in SPSS ver. 21.0. Program for the lethal time (LT_50_, KT_50_) values.

## Results

**Fig. 1.**
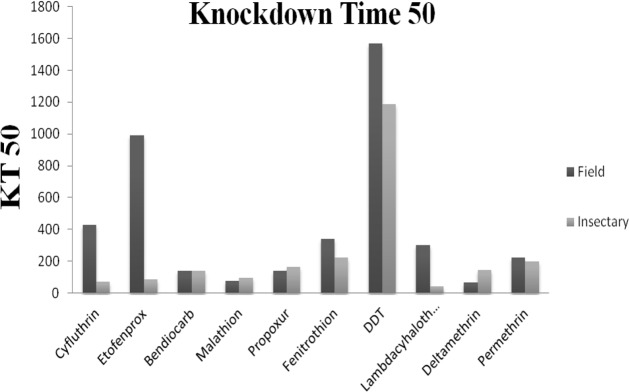
KT_50_ of field population compared to insectary strains of *Culex pipiens* to different insecticides

**Fig. 2.**
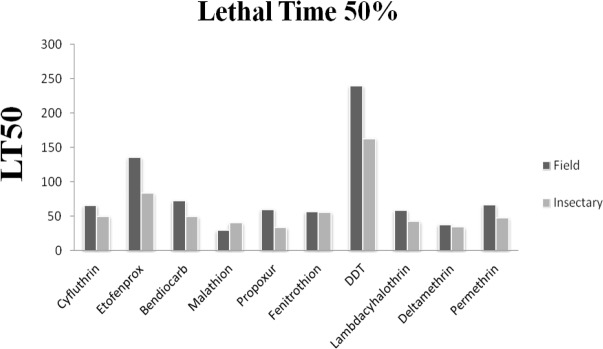
LT_50_ of field population compared to insectary strains of *Culex pipiens* to different insecticides

**Fig. 3.**
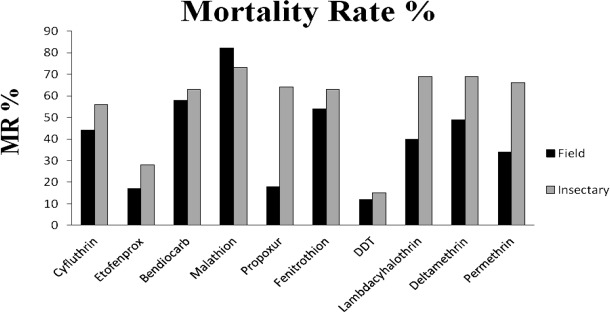
Mortality rate (%) of field population compared to insectary strains of *Culex pipiens* to different insecticides

**Fig. 4.**
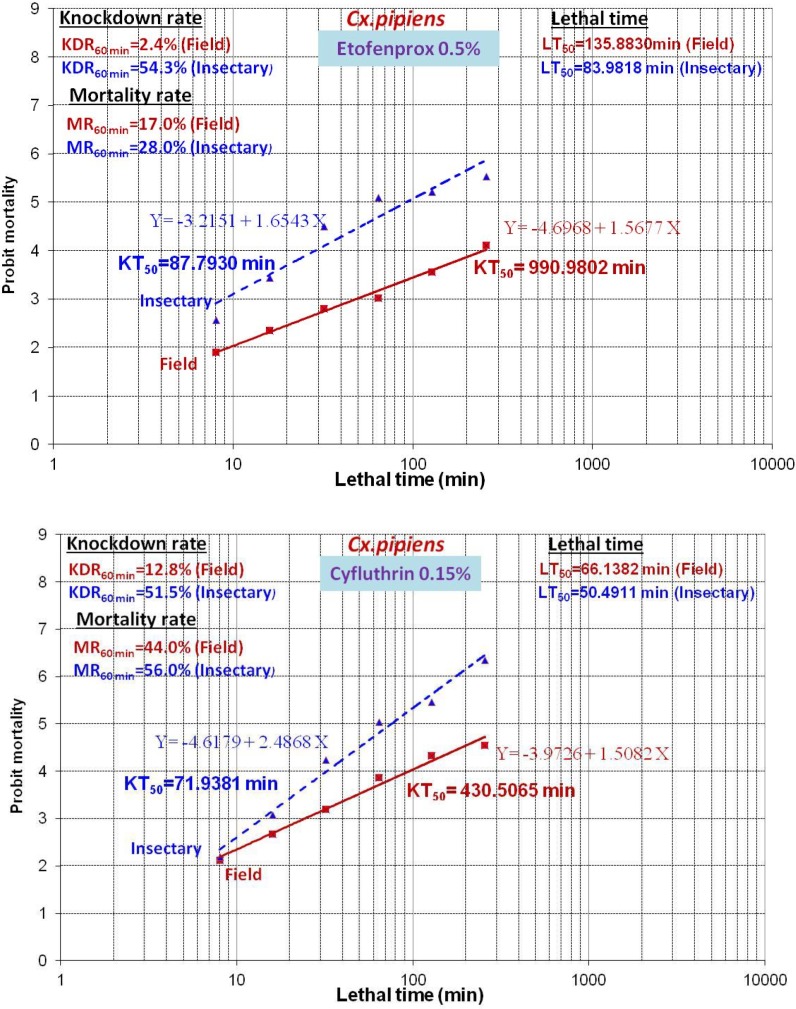

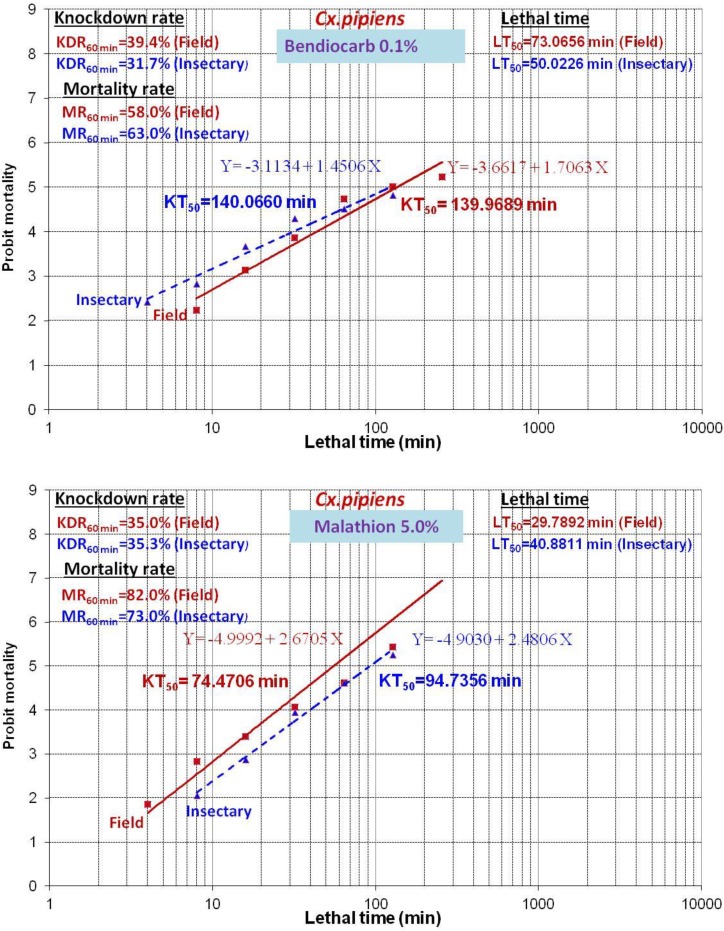

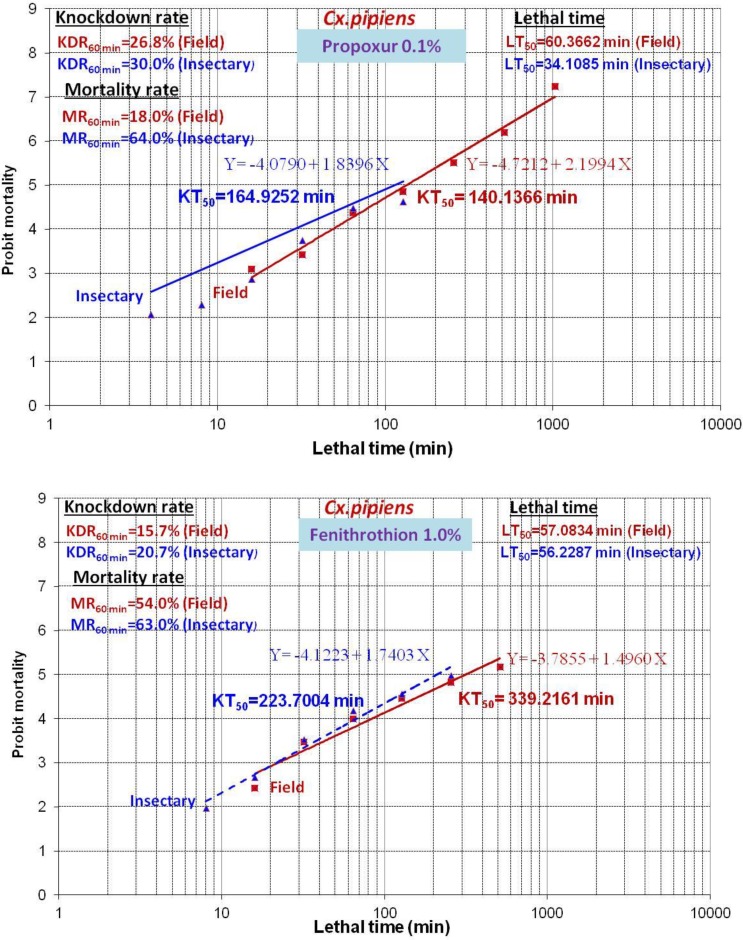

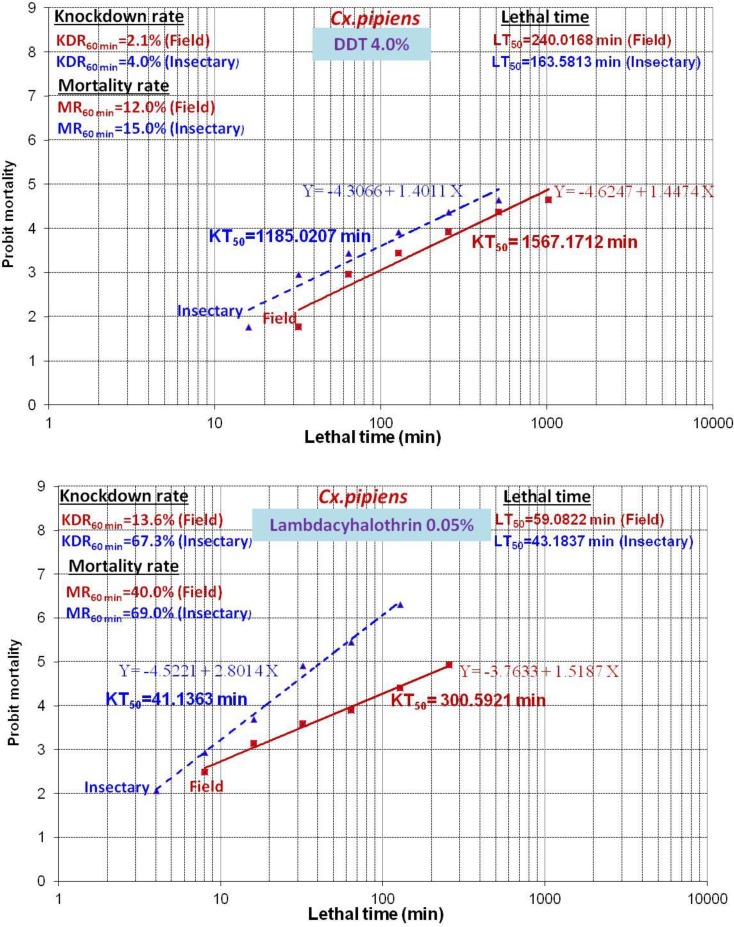

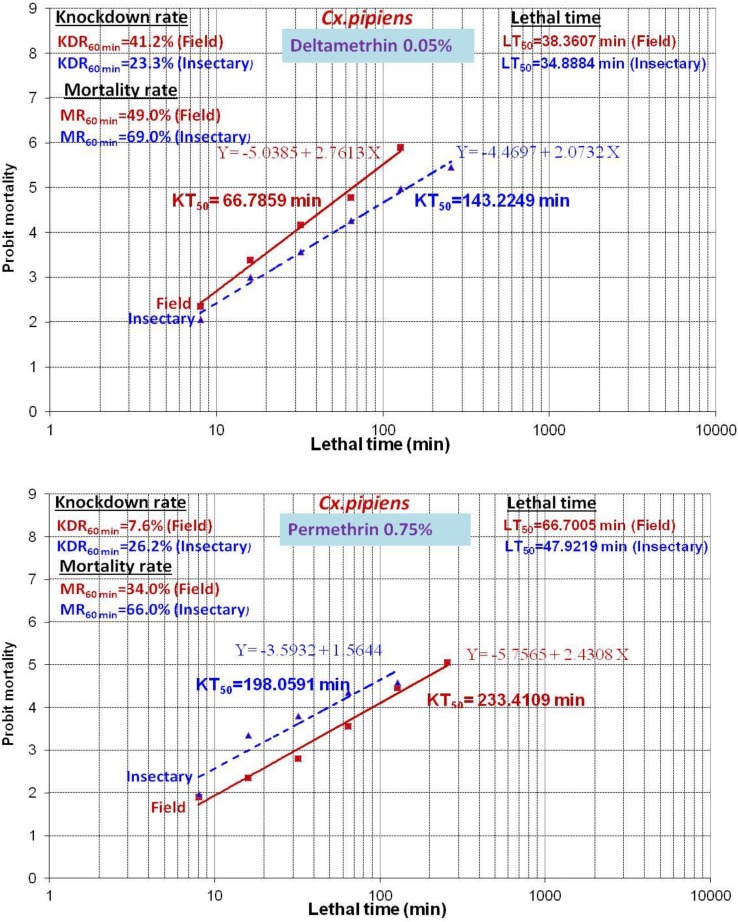
Comparison of knockdown with mortality indicators of adult *Culex pipiens* exposed to different insecticides

The results of the susceptibility test revealed that adults of *Cx. pipiens* from Tehran were highly resistant to mentioned insecticides (mortality rates under 90%) with the mortality rate recorded after one hour exposure followed by 24h recovery period are given in Table 1 and Figs. 3, 4. The comparison of mortalities between *Cx. pipiens* lab strain and field strain is shown in Figs. 3, 4. By applying WHO criteria (98–100% mortality indicates susceptibility, 90–97% mortality requires confirmation of resistance with other methods and <90% mortality suggests resistance) it was found that field strain is resistant to all of mentioned insecticides. Knockdown tests of adult female field and lab strains were performed by organochlorine, organophosphate, carbamate and pyrethroids insecticides in logarithm times of 8 to 256min. Overall, knockdown time 50% (KT_50_) of female *Cx. pipiens* complex of field strain and Lab strain of “Tehran University of Medical Sciences” are presented in Tables 1, Figs. 1, 4. In susceptibility tests with the same insecticides on female field and lab strains of *Cx. pipiens* complex, lethal time of 50% (LT_50_) in diagnostic dosages of insecticides are presented in Tables 1, Figs. 2, 4.

**Table 1.** Mortality rate, LT_50_, knockdown rate and KT_50_ of field and insectary strains of *Culex pipiens* to different insecticides

	**Field strain**					**Insectary strain**				

**Insecticides**	**DD (%)**	**MR (%)**	**LT_50_ (min)**	**KR (%)**	**KT_50_ (min)**	**DD (%)**	**MR (%)**	**LT_50_ (min)**	**KR (%)**	**KT_50_ (min)**
**Cyfluthrin**	0.15	44.0	66.0	12.8	430	0.15	56.0	50.4	51.5	71.9
**Etofenprox**	0.5	17.0	136	2.4	990	0.5	28.0	83.9	54.3	87.7
**bendiocarb**	0.1	58.0	73	39.4	139	0.1	63.0	50.0	31.7	140.0
**Malathion**	5.0	82	29.7	35	74.5	5.0	73.0	40.8	35.3	94.7
**Propoxur**	0.1	18	60.3	26.8	140.1	0.1	64.0	34.1	30.0	164.9
**Fenitrothion**	1.0	54.0	57.0	15.7	339.2	1.0	63.0	56.2	20.7	223.7
**DDT**	4.0	12.0	240.0	2.1	1567	4.0	15.0	163.5	4.0	1185
**Lambdacyhalothrin**	0.05	40.0	59.0	13.6	300.5	0.05	69.0	43.1	67.3	41.1
**Deltamethrin**	0.05	49.0	38.3	41.2	66.7	0.05	69.0	34.8	23.3	143.2
**Permethrin**	0.75	34.0	66.7	7.6	223.4	0.75	66.0	47.9	26.2	198

## Discussion

*Culex pipiens*, displayed resistance to mentioned insecticides that have been used by the local authorities for vector control program in field strain at the southern part of Tehran. In this study DDT resistant was observed in both laboratory and field collected strains of *Cx. pipiens*. Insecticide resistance levels of *Cx. pipiens* at the southern of Tehran have largely increased in the past several years (11). In this study, knockdown and susceptibility tests were conducted on 3–5 day old adult female field and lab strains sucrose-fed were exposed to the carbamate, organophosphate, organochlorine, and pyrethroid insecticides in logarithm times of 8 to 256min and 60 minutes, respectively. For the field strain, DDT had the lowest knockdown rate (2.1%) while deltamethrin insecticide had the highest rate (41.2%). Moreover, DDT insecticide had the lowest rate of knockdown (4.0%) for the field strain and lambdacyhalothrin insecticide had the highest rate of knockdown (67.3%). In this study, the highest mortality rate in the applied insecticides for the field strain was related to malathion (82.0%) and the lowest belonged to DDT (12.0%). For the lab strain, the highest mortality rate was related to malathion (73.0%) insecticide and the lowest belonged to DDT (15.0%). Knockdown and mortality rate values lower than 90% show the resistance of these mosquitoes against these insecticides (24, 25). In california, field and lab strains of *Cx. pipiens* complex species had a high tolerance against resmethrin, permethrin, etofenprox, and deltamethrin and knockdown rates for the field strain regarding these insecticides have been obtained as 5.0, 50.0, 45.0, and 50.0%, respectively (26). A study showed a high resistance of the field and lab strains of the *Cx. pipiens* complex to pyrethroid insecticides in california (27). High levels of *Cx. pipiens* complex resistance to pyrethroid insecticides were reported in Africa (28) and Asia (29, 30). A study performed about *Cx. pipiens* complex species had shown the high resistance of this species to DDT and pyrethroid insecticides. In addition, experiments performed in this study showed that the created high resistance is of the knockdown type in mosquitoes of this species. When knockdown resistance (Kdr) is observed in a species, the knockdown rate or amount can be considered as a sensitive indicator of early resistance detection (31). In china, the *Cx. pipiens* complex species showed high resistance to pyrethroid insecticides and the knockdown time (KT_50_) had also increased in his study which caused resistance against insecticides (32). In southern africa, study on important vectors of malaria (*Anopheles arabiensis*) showed a high resistance and increased knockdown time by 50% in this important vector against DDT, malathion, bendiocarb, dieldrin, deltamethrin, and permethrin (33). A knockdown and mortality rate of *Cx. pipiens* complex against 0.05% deltamethrin in exposure time of 60min in 5 regions of china was completely resistant to deltamethrin in four regions (Wei-fang, Jining, Jinan, Zibo). Knockdown rate during the exposure time of one hour was low and in qingdao region, this species showed tolerant to deltamethrin and knockdown rate and mortality rate were 41.90% and 83.81%, respectively (34). The required times for knockdown (KT_50_) were 241.6 and 255.8min for 0.05% lambda-cyhalothrin and 0.75% permethrin (in alrimaila region, 241.6 and 187.6min in arkaweet, 126.4 and 207.2min in burri, and finally 241.6 and 279.1min), respectively, in soba region in Sudan. The mortality rate of this species regarding the related insecticides was less than 90% which showed the complete resistance of this species in these regions (35, 36). In this study, our interpretations, as the fact that all field and lab strains (especially susceptible strain that is kept for several years in insectary without any exposure to pesticides) were resistant to tested insecticides, sometimes the added field mosquitoes to the insectary, do susceptibility test in an insectary, there is not special standard WHO susceptibility test of *culicinae* mosquitoes, are one of the reasons for this phenomenon.

## Conclusion

Extensive studies should be conducted to provide novel and better methods for more effective use of insecticides to protect the environment and reduce the concerns of both health and agriculture parts. Integrated approaches of vector control and determined resistance in mosquitoes should be regularly performed. Accordingly, teaching public health, especially to farmers regarding non-principled and excessive use of pesticides, should be considered. It would be useful if the insecticides are used on rotational basis to mitigate the selection pressure of insecticides against the vector species and increase the duration of the usage of the current insecticides. The data obtained from this study can be used in making timely management decisions about the judicious choice of insecticides in a vector control measures.
